# The relative age effect and its influence on athletic performance in Chinese junior female’ tennis players

**DOI:** 10.1371/journal.pone.0298975

**Published:** 2024-03-07

**Authors:** Yisheng Aku, Chengbo Yang

**Affiliations:** Department of Athletic Training, Chengdu Sport University, Chengdu, China; Instituto Politécnico de Santarém: Instituto Politecnico de Santarem, PORTUGAL

## Abstract

The relative age effect (RAE) has been the focus of numerous studies; however, there are still fewer studies in women’s sports than in men’s sports. In this study, all female players (*N* = 2,255) who participated in Chinese junior tennis competitions (U12, U14, U16, and the National Junior Team) from 2014 to 2019 were investigated in terms of competitors’ birth dates and year-end rankings. For the purposes of the analysis, the birth dates were also separated into quarters and half years. The study’s objectives were to analyze the prevalence of the RAE among young Chinese female tennis players and to further examine how the RAE affects athletic performance. Differences between the observed and expected distributions of birth dates were tested using the chi-square statistic, and subsequent calculations were tested using odds ratios. The RAE was discovered to be present in every group of Chinese junior female tennis players (*p* < 0.001), with the proportions of those born in the first half of the year being 56.4% (U12), 53.1% (U14), and 57.0% (U16), respectively. The RAE for athletes selected for the National Women’s Junior Tennis Team was even more significant, with the percentage of birth dates in the first half of the year at 61.2%. Finally, we observed a tendency for the effect of the RAE on the athletic performance of adolescent female tennis players to diminish with age.

## Introduction

The relative age effect (RAE) has been studied intensively over the year. The RAE is a statistical bias observed across sports contexts and consists of a systematic skewness in birth date distribution within an annual age cohort [1]. The RAE is interpreted in sport science research to mean that athletes who are born earlier in a given selection or competition year enjoy advantages in physical, emotional, and cognitive development as well as performance, and they are more likely to be chosen for higher-level sports teams and talent development programs than those who are born later in the same year [2]. As a result, relatively older athletes can be favored in their divisions for talent identification, selection, and development and are more likely to have better development opportunities.

The earliest research report on the RAE was in the field of education [3], while in the field of sports, Grondin et al. [4] first observed the RAE in hockey, and Barnsley et al. [2] discovered an RAE in sports during the same period. The RAE has been repeatedly discovered in sports over the past 10 years of research, including in basketball [5, 6], soccer [1, 7, 8], volleyball [9, 10], and tennis [11]. Moreover, reports on the impact of the RAE have been published with regard to sports [2, 12, 13], education systems [14–16], specific medical diagnoses [17, 18], and cognitive tasks [19].

The RAE reflects the advantage (disadvantage) created by the interaction between an athlete’s birth date and the yearly selection cutoff date. Because of this gap, relatively older children enjoy maturational, physical, cognitive, and emotional advantages over their younger peers in the same age group [20–22]. Various RAE-triggering mechanisms have been explored in numerous studies, and theories supporting the existence of RAEs include the mature selection hypothesis, the Matthew effect, the Pygmalion effect, and the Galatea effect. The Matthew effect is exemplified by the fact that the rich get richer and the poor get poorer; the Pygmalion effect can be summarized as the greater the expectations of a person, the greater the outcome that person will achieve; and the Galatea effect is explained by the fact that once expectations are imposed on a person, that person’s behavior is usually consistent with those expectations [23, 24]. Thus, the RAE unfairly affects young athletes’ ability to develop and may cause late-born athletes to give up their sport altogether [25–27], resulting in a huge talent drain. There has also been a fair amount of discussion about RAE-triggering mechanisms in sports. Specifically, the triggering of RAEs is multifactorial and mixed and depends on factors such as age group and position in the game [7], socio-cultural context [28], level of competition [5], and historical moments [1]. As a result, the RAE has emerged as one of the most important and pervasive elements that may influence bias in talent identification and selection in sports [29–31]. Given the pressure for short-term results, players with earlier birth dates may often have a greater chance of being selected, leading to more incentives and real opportunities [32].

In this regard, in tennis, regardless of the season of birth, all players should have an equal opportunity to participate in competitive tennis activities. Nevertheless, most of the research results still show the prevalence of the influence of birth date on tennis players. In two of the first RAE studies on tennis [33, 34], it was discovered that there were typically more athletes born in the first part of the year than in the second. Half of the top Dutch tennis players between the ages of 12 and 16 were born in the first quarter of the year (according to Dudink [33]), while 85% of British junior-high-level tennis players were born between January and June (according to Baxter-Jones [34]). The influence of the calendar year on tennis stems from the fact that since 1995, junior events have had a January 1 cutoff date set by the International Tennis Federation (ITF). Players in the same age group may range in actual age by up to 24 months because junior tennis divisions are divided into two-year age groups, which exacerbates the degree to which the RAE occurs in tennis. In a study of top tennis players, it was found that more than half of the top 100 players were born in the first semester, both in terms of individual years (53.0–63.0%) and the entire observed period (58.4%) [11]. In several other studies in tennis, the RAE was found to be present in adolescent male tennis players (U14 and U16) [35], adolescent female tennis players (15–18 years old) [36], and athletes born in 1995–2000 [37]. In contrast, only a small RAE was found among Swedish junior male and female tennis players born in 1998–2001 [38].

A relatively small portion of the many RAE studies focus on women. The RAE occurs to a smaller extent in women’s sports than in men’s sports, and this may be due to a number of factors. The RAE is infrequently observed in female athletes, according to a meta-review that also revealed that the variability of the RAE in women’s sports may be influenced by a number of interrelated restrictions [31]. In this regard, the reasons for the RAE being different in women’s sports versus men’s may also be multifactorial, with girls entering puberty earlier than boys, boys reaching their maximum altitude speed almost two years later than girls [39], and girls having slightly less marked physical differences than boys [40, 41]. Moreover, when most sports are under the highest selection pressure, girls’ puberty has frequently passed, and the differences brought on by various stages of physical development are not readily apparent [42, 43]. This has led to research findings reflecting that RAEs were different for girls versus boys in some sports and that RAEs did not exist in some women’s sports, as the periods of intense selection and the periods when the difference in physical development between boys and girls is highest did not match. For instance, in women’s soccer, neither the German women’s national team [8] nor the Swiss elite women’s soccer team [44] exhibited any RAE. The US women’s soccer team’s RAE is also minimal [45], and road cycling has not been linked to any RAEs [46].

Based on the above analysis, the RAE in youth sports can result in a significant talent drain, which exists in different forms in men’s and women’s sports. Moreover, there is no relevant literature analyzing the existence of the RAE among Chinese female tennis players or its impact on athletic performance. To improve the success rate of tennis players and reduce the talent drain, it is crucial to examine whether a problem exists before taking targeted measures. Therefore, it is necessary to investigate the presence of the RAE and its impact on athletic performance among Chinese junior female tennis players. Accordingly, the purpose of this study is to reveal the extent of RAE occurrence among Chinese junior female tennis players to further reveal the effect of the RAE on sport performance acquisition in this group.

## Methods

### Participants

Athletes participating in Chinese junior tennis age-category tournaments are the most representative group of junior athletes in China; consequently, any study of the occurrence of RAEs among Chinese junior female athletes must include this group. For this reason, we included all Chinese junior female athletes with tennis point rankings in the study. We selected all female tennis players with year-end point rankings (*N* = 2,152) who participated in China’s junior tennis age-category tournaments (U12, U14, and U16) from 2014 to 2019, as well as those who had been selected for the National Junior Tennis Team and Junior Reserve Team lists in 2019 and 2020 (*N* = 103). The distribution of birth years and months and the relationships between year-end rankings and RAEs were analyzed for these study subjects.

The abovementioned athletes’ birth dates and athletic performance were obtained from the China Tennis Association’s official website (www.tennissport.org.cn). Data extraction included each player’s name, birth date, and year-end ranking results. Due to the study data being in the public domain, no informed consent or approval by an ethics committee was required.

### Statistical analysis

Based on the birth dates, each player was assigned to one of four respective age quarters (Q) and two semesters (S) to calculate the RAEs of the different age categories [47]. The cut-off date for the Junior Competition Grouping for both the ITF and the Chinese Tennis Association (CTA) is January 1 of each year. Therefore, the year was divided into quarters: Q1 = January, February, and March; Q2 = April, May, and June; Q3 = July, August, and September; and Q4 = October, November, and December. Furthermore, the year was also divided into semesters: S1 = January to June and S2 = July to December for half-year distributions.

To examine the RAE, the distribution of the participants’ birth months across Q1–Q4 was examined using the chi-square (χ^2^) goodness-of-fit test. For the purposes of statistical analysis, the expected frequency of births in a quartile was set at 25%, assuming a homogeneous sample distribution [48]. The effect size was set at an alpha level of *p* < 0.05 for statistical significance and *p* < 0.01 for a highly significant differences. Odds ratios (ORs) with 95% confidence intervals (95% CIs) were calculated for Q1–Q4 [47]. The OR comparisons were interpreted as follows: OR < 1.22 was negligible, 1.22 ≤ OR < 1.86 was small, 1.86 ≤ OR < 3.00 was medium-sized, and OR ≥ 3.00 was large [49]. All statistical analyses were performed using SPSS 25.0 (USA).

## Results

### Relative age effect on the women’s U12 category (2014–2019)

As can be seen in Table 1, the percentages of births in the first to fourth quarters for the total U12 female category from 2014–2019 were 33.5%, 22.9%, 24.4%, and 19.2%, respectively, and in the first and second half of the year, they were 56.4%, and 43.6%, respectively, with a chi-square value (χ^2^) of 38.215 (*p* < 0.001). This represents overrepresentation of relatively older peers in U12 female tennis players.

**Table 1:** Distribution and analysis of the birth dates of female U12 athletes from 2014–2019.

Year	Classify	Birthdate distribution					Semesters		Data analysis		
		Q1	Q2	Q3	Q4	Total	S1	S2	X^2^	*p*	OR
**2014**	n	34	18	19	22	93	52	41	7.000	.072	1.55
	%	36.6	19.4	20.4	23.7	100.0	56.0	44.0			
**2015**	n	34	22	20	17	93	56	37	7.172	.067	2.00
	%	36.6	23.7	21.5	18.3	100.0	60.2	39.8			
**2016**	n	36	22	28	12	98	58	40	12.531	.006**	3.00
	%	36.7	22.4	28.6	12.2	100.0	59.2	40.8			
**2017**	n	32	19	22	19	92	51	41	4.957	.175	1.68
	%	34.8	20.7	23.9	20.7	100.0	55.5	44.5			
**2018**	n	36	25	25	17	103	61	42	7.097	.069	2.12
	%	35.0	24.3	24.3	16.5	100.0	59.3	40.7			
**2019**	n	115	90	95	77	377	205	172	7.923	.048*	1.49
	%	30.5	23.9	25.2	20.4	100.0	54.4	45.6			
**Total**	n	287	196	209	164	856	483	373	38.215	< .001	1.75
	%	33.5	22.9	24.4	19.2	100.0	56.4	43.6			

Q1–Q4: The first to the fourth quarter, X^2^: chi-square value, *p* < 0.05: significant difference*, *p* < 0.01: very significant difference**, OR: ratio of Q1 and Q4.

Overall, there was a significant relative age effect among Chinese U12 women’s tennis players. S1/S4 (OR) values showed a significant effect in all years, with OR values as high as 3.0 in 2016 and as low as 1.49 in 2019. Although certain years did not differ substantially in terms of *p*-values, all years showed a significant effect in terms of S1/S4 (OR) values. Overall, the skewed distribution of birth dates for the entire U12 cohort was the most severe, with the number of births in the first quarter and the first half of the year being significantly higher than those in the fourth quarter and the second half of the year, revealing a prevalence of RAEs among U12 female tennis players in China. Looking at the 2014–2019 period, the RAE for U12 female tennis players ebbs and flows over these years, and while it increases in the first few years, there is no clear pattern.

### Relative age effect on the women’s U14 category (2014–2019)

As can be seen in Table 2, the percentage of births in the first through fourth quarters for the entire U14 girls’ cohort from 2014–2019 were 31.0%, 22.1%, 24.6%, and 22.3%, respectively, while 53.1% and 46.9% of the birth dates were in the first and second halves of the year, respectively, with a chi-square value (χ^2^) of 14.944 (*p* < 0.01). This represents overrepresentation of relatively older peers.

**Table 2:** Distribution and analysis of the birth dates of female U14 athletes from 2014–2019.

Year	Classify	Birthdate distribution					Semesters		Data analysis		
		Q1	Q2	Q3	Q4	Total	S1	S2	X^2^	*p*	OR
**2014**	n	37	28	30	31	126	65	61	1.429	.699	1.23
	%	29.4	22.2	23.8	24.6	100.0	51.6	48.4			
**2015**	n	31	22	23	18	94	53	41	3.787	.285	1.72
	%	33.0	23.4	24.5	19.1	100.0	56.4	43.6			
**2016**	n	37	20	27	19	103	57	46	8.029	.045*	1.95
	%	35.9	19.4	26.2	18.4	100.0	55.3	44.7			
**2017**	n	36	25	25	18	104	61	43	6.385	.094	2.00
	%	34.6	24.0	24.0	17.4	100.0	60.6	39.4			
**2018**	n	32	26	26	24	108	58	50	1.333	.712	1.33
	%	29.6	24.1	24.1	22.2	100.0	53.7	46.3			
**2019**	n	48	36	44	49	177	84	93	2.367	.500	0.98
	%	27.1	20.3	24.9	27.7	100.0	47.4	52.6			
**Total**	n	221	157	175	159	712	378	334	14.944	.002**	1.39
	%	31.0	22.1	24.6	22.3	100.0	53.1	46.9			

Q1–Q4: The first to the fourth quarter, X^2^: chi-square value, *p* < 0.05: significant difference*, *p* < 0.01: very significant difference**, OR: ratio of Q1 and Q4.

Among the U14 female tennis players, only the year 2014 had a negligible effect in terms of S1/S4 (OR) values, while all other years had varying degrees of effect. The degree of skewness of the distribution of dates of birth was significant (*p* < 0.01) across the U14 junior group, and although the effect was small in terms of OR, the proportion of birth dates in the first half of the year remained high at 53.1%. Therefore, the RAE phenomenon is still evident among U14 Chinese junior female tennis players. In the 2014–2019 period, there was a trend for the RAE in U14 female tennis players to become more severe and then gradually less severe, with less of an impact in the later years.

### Relative age effect on the women’s U16 category (2014–2019)

As shown in Table 3, of the total number of U16 female athletes in 2014–2019, there were 57.0% and 43.0% birth dates in the first and second halves of the year, respectively, and 33.2%, 23.8%, 21.2%, and 21.7% of the birth dates occurred in the first to fourth quarters, respectively, with a chi-square value (χ^2^) of 21.904 (*p* < 0.001). This indicates an overrepresentation of relatively older peers in U16 category.

**Table 3:** Distribution and analysis of the birth dates of female U16 athletes from 2014–2019.

Year	Classify	Birthdate distribution					Semesters		Data analysis		
		Q1	Q2	Q3	Q4	Total	S1	S2	X^2^	*p*	OR
**2014**	n	39	28	25	22	114	67	47	5.789	.122	1.77
	%	34.2	24.6	21.9	19.3	100.0	58.8	41.2			
**2015**	n	44	28	22	22	116	72	44	11.172	.011*	2.00
	%	37.9	24.1	19.0	19.0	100.0	62.0	38.0			
**2016**	n	30	24	27	23	104	54	50	1.154	.764	1.30
	%	28.8	23.1	26.0	22.1	100.0	51.9	48.1			
**2017**	n	23	17	17	17	74	40	34	1.459	.692	1.35
	%	31.0	23.0	23.0	23.0	100.0	54.0	46.0			
**2018**	n	29	22	19	23	93	51	42	2.269	.519	1.26
	%	31.2	23.7	20.4	24.7	100.0	54.9	45.1			
**2019**	n	29	20	14	20	83	49	34	5.530	.137	1.45
	%	34.9	24.1	16.9	24.1	100.0	59.0	41.0			
**Total**	n	194	139	124	127	584	333	253	21.904	< .001	1.53
	%	33.2	23.8	21.2	21.7	100.0	57.0	43.0			

Q1–Q4: The first to the fourth quarter, X^2^: chi-square value, *p* < 0.05: significant difference*, *p* < 0.01: very significant difference**, OR: ratio of Q1 and Q4.

For the older age category in junior girls’ tennis (U16), only the year 2018 had a negligible impact in terms of S1/S4 (OR) values. All the other years had varying degrees of impact, exerting a smaller impact overall. In the entire U16 cohort, the skewness of the distribution of athletes’ birth dates was significant (*p* < 0.01), with 57.0% born in the first half of the year. Furthermore, a significant RAE was observed in the cohort. In the 2014–2019 period, the RAE of the U16 female tennis player category resembled that of the U12 category, with no clear trend and a wavelike pattern. Notably, the impact was more pronounced in the earlier years.

### Relative age effect on the Chinese National Women’s Junior Tennis Team

As indicated in Table 4, the percentage of birth dates of athletes selected for the National Women’s Junior and Reserve Teams in 2019 and 2020 were 34.0%, 27.2%, 23.3%, and 15.5% in the first through fourth quarters, respectively, and 61.2% and 38.8% in the first and second halves of the year, respectively, with a chi-square value (χ^2^) of 7.330 (*p* > 0.05). Although there is no significant difference from the chi-square value, the OR effect value is as high as 2.19, which is a medium effect value, and the proportion of athletes born in the first half of the year is also very high (61.2%). Thus, the RAE was also evident in the Chinese National Women’s Junior Tennis Team.

**Table 4:** Distribution and analysis of Chinese national female junior tennis team.

Year	Classify	Birthdate distribution					Semesters		Data analysis		
		Q1	Q2	Q3	Q4	Total	S1	S2	X^2^	*p*	OR
**2019**	n	11	8	10	4	33	19	14	3.485	.323	2.75
	%	33.3	24.2	30.3	12.1	100.0	57.6	42.2			
**2020**	n	8	8	4	4	24	16	8	2.667	.446	2.00
	%	33.3	33.3	16.7	16.7	100.0	66.7	33.3			
**2020 Reserves**	n	16	12	10	8	46	28	18	3.043	.385	2.00
	%	34.8	26.1	21.7	17.4	100.0	60.9	39.1			
**Total**	n	35	28	24	16	103	63	40	7.330	.062	2.19
	%	34.0	27.2	23.3	15.5	100.0	61.2	38.8			

Q1–Q4: The first to the fourth quarter, X^2^: chi-square value, *p* < 0.05: significant difference*, *p* < 0.01: very significant difference**, OR: ratio of Q1 and Q4.

### Relative age effect on the performance of young female tennis players

In order to better compare the effects of the RAE on different sport performances in Chinese women’s junior tennis, we divided the athletes in each age category into the following groups: the entire cohort comprised of all players, the top 30 ranked players, and the bottom 30 ranked players.

**Fig 1:**
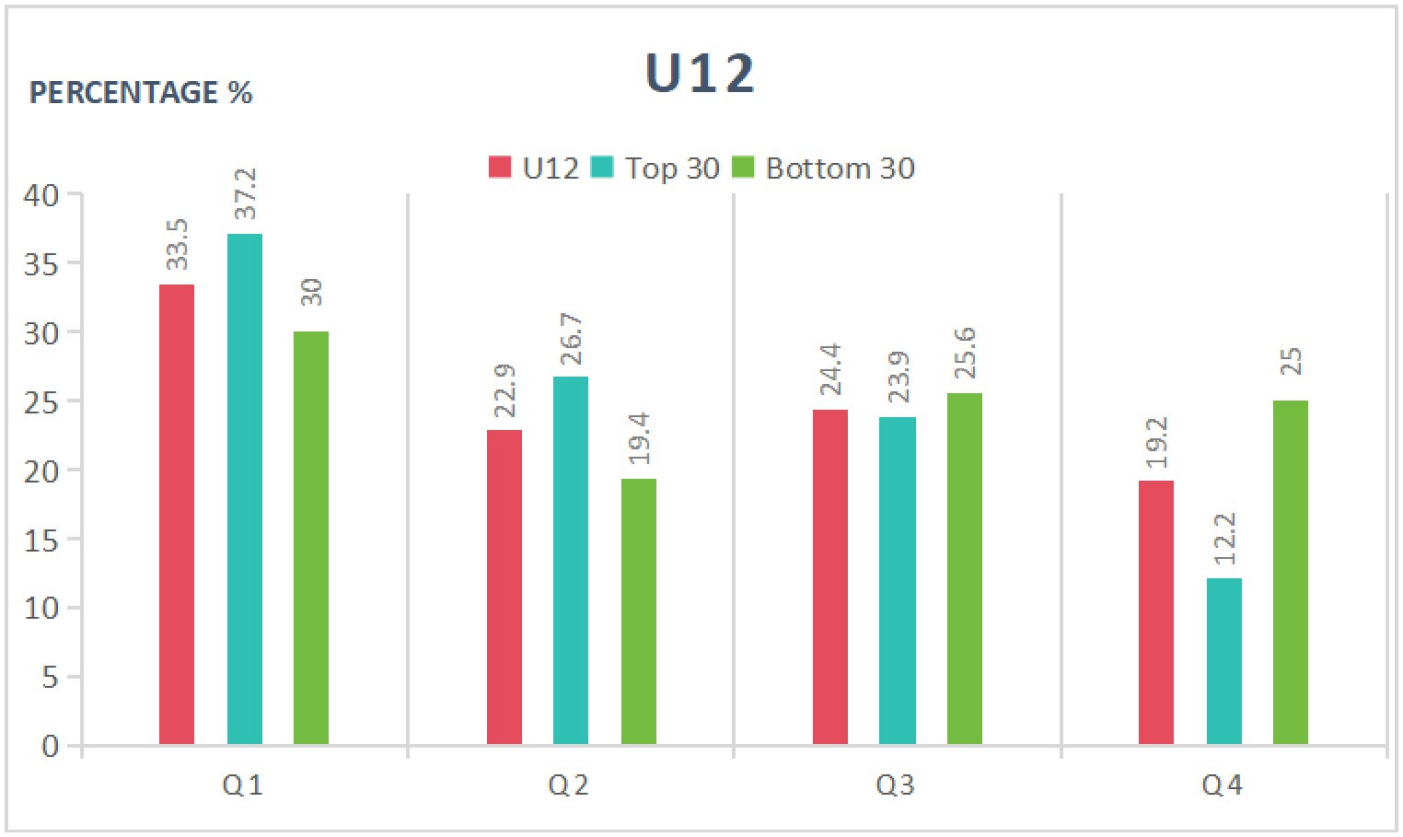
Distribution of birth quartile in the U12 group considering different sport performance ranking.

**Fig 2:**
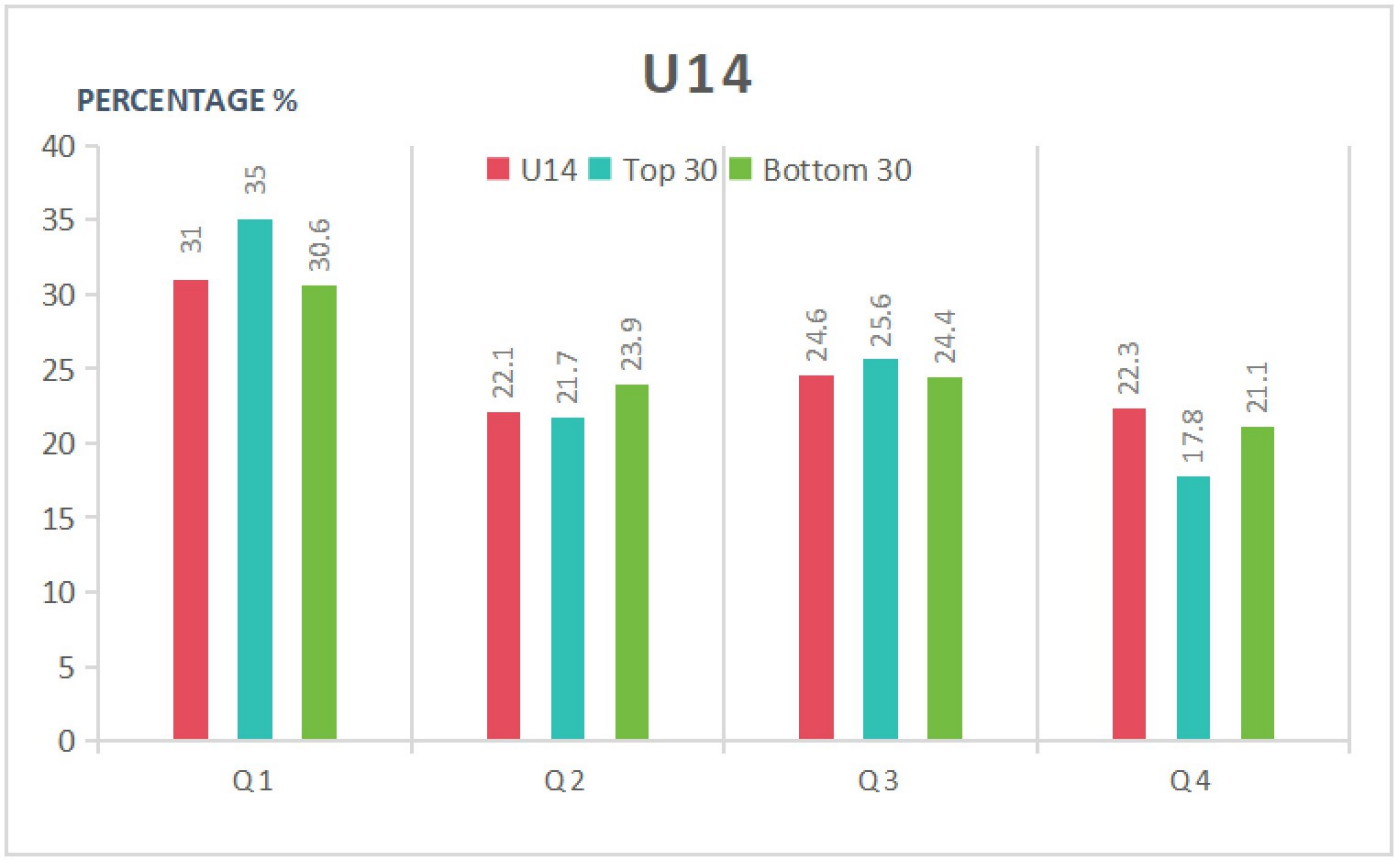
Distribution of birth quartile in the U14 group considering different sport performance ranking.

**Fig 3:**
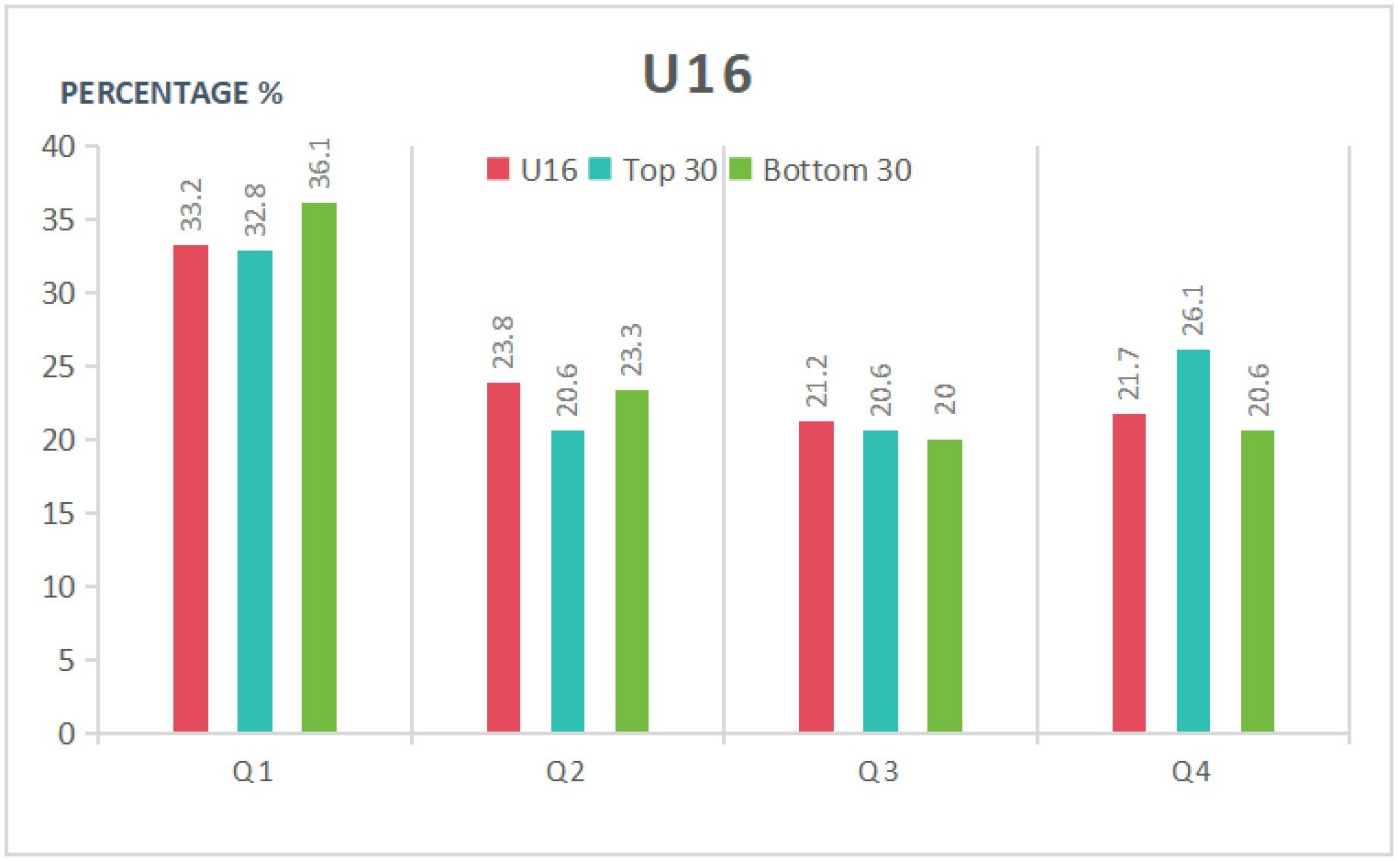
Distribution of birth quartile in the U16 group considering different sport performance ranking.

As can be seen in Table 5 and Figs 1–3, in the U12 category, the proportion of birth dates in the first quarter was higher among the top 30 players than in the entire cohort or the bottom 30 group; furthermore, the proportion of birth dates in the fourth quarter was lower than in the entire cohort or the bottom 30 group. The top 30 group exhibited a 1.88 higher OR, a 14.5% greater incidence of birth dates in the first half of the year, and a 7.2% greater incidence of birth dates in the first quarter compared to the bottom 30 athletes. Statistically significant differences were found in the distribution of dates of birth for the top 30 group, while no significant differences were found for the bottom 30. Overall, the RAE had a significant impact on the top-ranked female tennis players in the U12 age category and a negligible impact on the bottom-ranked players. Moreover, the athletes’ athletic performance was more likely to improve the closer their birth date was to the start of the year.

**Table 5:** Comparison of the RAE in top 30, bottom 30 and overall female athletes (2014–2019).

Group	Classify	Birthdate distribution					Semesters		Data analysis		
		Q1	Q2	Q3	Q4	Total	S1	S2	x^2^	*p*	OR
**U12**	n	287	196	209	164	856	483	373	38.215	< .001	1.75
	%	33.5	22.9	24.4	19.2	100.0	56.4	43.6			
**U12-Top 30**	n	67	48	43	22	180	115	65	22.800	< .001	3.05
	%	37.2	26.7	23.9	12.2	100.0	63.9	36.1			
**U12-Bottom 30**	n	54	35	46	45	180	89	91	4.044	.257	1.17
	%	30.0	19.4	25.6	25.0	100.0	49.4	50.6			
**U14**	n	221	157	175	159	712	378	334	14.944	.002**	1.39
	%	31.0	22.1	24.6	22.3	100.0	53.1	46.9			
**U14-Top 30**	n	63	39	46	32	180	102	78	11.778	.002**	1.97
	%	35.0	21.7	25.6	17.8	100.0	56.7	43.3			
**U14-Bottom 30**	n	55	43	44	38	180	98	82	3.422	.331	1.45
	%	30.6	23.9	24.4	21.1	100.0	54.4	45.6			
**U16**	n	194	139	124	127	584	333	253	21.904	< .001	1.53
	%	33.2	23.8	21.2	21.7	100.0	57.0	43.0			
**U16-Top 30**	n	59	37	37	47	180	96	84	7.289	.063	1.26
	%	32.8	20.6	20.6	26.1	100.0	53.3	46.7			
**U16-Bottom 30**	n	65	42	36	37	190	107	73	12.311	.006**	1.76
	%	36.1	23.3	20.0	20.6	100.0	59.4	40.6			

Q1–Q4: the first to the fourth quarter, X^2^: chi-square value, *p* < 0.05: significant difference*, *p* < 0.01: very significant difference**, OR: ratio of Q1 and Q4.

In the U14 age category, the top 30 group again had a higher proportion of birth dates in the first quarter but a lower proportion in the fourth quarter than the entire cohort or the bottom 30 group. The proportion of athletes in the top 30 group with a birth date in the first quarter was 4.4% higher than those in the bottom 30 group and 4% higher than the entire U14 cohort. The proportion of athletes born in the first half of the year in the U14 category was relatively similar for the three groups, with smaller gaps between them compared to the U12 category. Overall, the effect of date of birth on athletic performance was less significant in the U14 female category than in the U12 category, with relatively equal opportunities for athletes born in all quarters.

Unlike the two categories analyzed above, in the U16 category, the proportion of the top 30 athletes born in the first quarter was lower than in the entire cohort or the bottom 30, and for the fourth quarter, the proportion of the top 30 athletes was higher than in the entire cohort or the bottom 30. In contrast, the bottom 30 athletes had a higher percentage of birth dates in both the first quarter and the first half of the year than the entire cohort or the top 30 group, and a lower percentage than the entire cohort or the top 30 group in the fourth quarter and the second half of the year. A comparison of S1/S4 (OR) values shows that the values for both the top 30 and the bottom 30 are lower than the value for the entire cohort, with less fluctuation in the percentages of the different groups in each quarter than in the U12 or U14 age categories. Overall, the effect of the RAE on the athletic performance of U16 female tennis players was different from the groups analyzed above, with no significant effect on either the top- or bottom-ranked players.

## Discussion

This study’s major goal was to investigate whether the RAE exists in Chinese junior women’s tennis. We compared and analyzed the birth dates of athletes with various levels of achievement within the same age categories and investigated the impact of birth dates on athletic performance. The RAE was found to be present in all groups of Chinese junior female tennis players, and the observations were consistent with those found in some other female sports [50, 51] and in tennis [11, 35–38]. Our study identified the presence of the RAE for the first time in Chinese junior female tennis, providing a foundation for further research on RAE mitigation programs and other studies in Chinese youth sports.

There was a significant RAE in all age categories in Chinese junior female tennis. A skewed distribution of birth dates was identified for each category, and the difference was statistically significant (*p* < 0.05). The percentages of the total number of players in each group whose date of birth was in the first quarter were 33.5% (U12), 31.0% (U14), and 33.2% (U16), and the percentages of those whose date of birth was in the first half of the year were 56.4% (U12), 53.1% (U14), and 57.0% (U16). It is difficult to see a clear trend of increasing or decreasing RAE from the percentage of birth dates. However, an effect of date of birth on sports performance is only present in the younger age groups; it becomes less significant as the players get older, as can be seen from the subgroup sports performance comparisons. Date of birth had no effect on athletic performance even in the U16 age group, and similar results in female sports [31] are consistent with the study’s finding that the impact of the RAE on athletic performance declines with age. This is determined by a number of factors. Relatively older players in younger age categories may have greater physical, technical, mental, and behavioral advantages [32]. Furthermore, female athletes have an earlier physiological surge in growth than male athletes, and the earlier developmental start allows most female athletes to reach above-average physical maturity in the older age categories. Due to this developmental advantage in the older age categories, an earlier date of birth no longer has an impact on athletic performance.

Several authors have observed that the RAE in female sports decreases as the level of competition increases and that this is relatively more pronounced in French and U.S. soccer and handball in Germany [12, 50, 52]. Some studies have interpreted this phenomenon of declining RAE in women as an interaction between biological and maturational differences on the one hand and social influences on the other [45]. It has also been suggested that this is due to technical, tactical, and mental skills becoming more important in the selection process [53, 54]. The occurrence of this phenomenon among Chinese female athletes may also be attributed to pressures from socially constructed gender roles, which may cause precocious females to lack the motivation to achieve at a high level because Chinese society does not value the achievements of female athletes as much as those of male athletes [55].

Meanwhile, the presence of the RAE was more pronounced for the Chinese National Women’s Junior Tennis Team than for other categories, with ORs as high as 2.75 in some years. This may be because the more rigorous selection process favors the play of relatively older players, which leads to a larger observed RAE. In addition, the selection criteria for China’s junior tennis teams when organizing training camps are based on the results of competitions at all levels, which undoubtedly increases the space for more mature athletes to play, leading to an RAE that is generally more pronounced in China’s national junior teams than in other groups.

As a highly commercialized sport, tennis is played more than many other sports during the adolescent years, which undoubtedly increases the probability of RAEs in the sport, as the overly competitive environment during these years is a significant contributor to the development of RAEs. Like the present study, many other tennis studies have found that the RAE is present in tennis and has an impact on youth tennis performance. In their study of U.S. junior tennis players, Giacomini et al. [35] discovered the prevalence of the RAE and a strong relationship between date of birth and athletic performance in male athletes in the U14 and U16 age categories. However, among girls, the effect of date of birth on tennis rankings was not significant in any age group. The RAE was discovered in Swedish tennis players born between 1998 and 2001 by Gerdin [38], with an average of 64.1% of the top 10 players born in the first half of the year. These findings are comparable to those of the current study in many ways.

While there is no doubt that athletes born throughout the year should receive a fair chance, regardless of whether they are born in the first or fourth quarter, the reality is that the RAE continues to be prevalent in sports, and fairness in this regard is still substantially difficult to achieve. There are many reasons for this unfairness, such as the Matthew, Pygmalion, and Galatea effects [23, 24], which we have already mentioned. Against this backdrop, many studies have explored ways to mitigate the RAE, such as replacing calendar age with physiological age [56, 57], changing or rotating selection dates [58, 59], and improving coaches’ evaluations of talented players [58, 60, 61]. One study proposed letting coaches keep younger players at the junior level. While this negatively affects individual and team performance at a younger age, it may favor long-term success [62]. In contrast, no studies on methods to mitigate the RAE have been seen in current research in China.

Our study revealed that among Chinese junior female tennis players, the correlation between achieved athletic performance and date of birth decreases with age. This indicates that older Chinese junior female tennis players no longer have a relatively easy time improving their athletic performance the closer their birth date is to the start of the year, which may be connected to the fact that female athletes have an earlier physiological development period than male athletes. However, at the same time, the RAE is still evident in the overall female U14 and U16 categories, which may be related to the participation of younger age female athletes in earlier developmental stages. Athletes with later birth dates fail to perform well at younger ages and drop out of the sport, resulting in a skewed age distribution that continues to be transmitted to the older age groups. This can be explained by the Matthew effect (the rich get richer and the poor get poorer) [23, 24]. Based on this, it has been suggested that for relatively younger players, long-term development should be facilitated to confer a significant potential for success at the adult level, including the enhancement of, for example, technical and tactical skills, as well as superior psychological and social skills necessary to overcome the odds of the RAE [63–66].

Creating a fair and sustainable environment for the development of sports and reducing the talent drain are aspirations of many who are committed to promoting the sustainable development of sports. At the same time, we should be aware that the physiological, kinanthropometric, conditional, and psychological profiles of players should be understood as a framework for guiding the development of athletes’ talent, but not as a selection tool [67]. Therefore, based on the finding that the RAE exists in Chinese junior female tennis, we suggest that Chinese junior tennis management organizations, coaches, athletes, and athletes’ parents should be fully aware of the negative effects of the RAE on all aspects of athletes’ growth and development. In the process of selecting and cultivating athletes, there should be less examination of the existing sports performance of young athletes and more emphasis on the development of athletes’ potential ability to minimize the temporary disadvantage of late-born athletes. Secondly, when organizing junior tournaments, the CTA should mitigate the negative impact of the RAE by reforming the grouping designations of junior tournaments and the cut-off dates for rotating competition groupings. Finally, as Simon et al. [68] suggested, coaches need to retain all players to some extent and avoid selecting only the most athletically accomplished so that late-born athletes can have the same opportunities to train and compete as early-born athletes. We suggest that Chinese tennis coaches, when training and organizing their teams, should not limit themselves to the performance of junior athletes in terms of physical morphology indicators and so on, but should give full consideration to athletes born in different years and times of the year.

Our study has some limitations. Firstly, we do not know the distribution of the dates of birth across the wider population of China. Secondly, although the results of this study will help to expand the knowledge of coaches, players, and tennis officials regarding RAE issues, we have not been able to make further recommendations based on the research of this study but rather only based on the methods of other researchers. Finally, only quarterly data were used in our study to analyze the impact of the RAE, which may inhibit our complete knowledge of the RAE problem.

## Conclusion

This is the first time that RAE in Chinese junior female tennis has been discussed and analyzed in a scholarly paper. The study found a skewed distribution of birth dates for all categories of athletes, with a significantly higher percentage of athletes born in the first quarter than in the fourth quarter, and a significantly higher percentage born in the first half of the year than in the second half of the year. The RAE was discovered to be present in all age categories of Chinese junior female tennis players. Moreover, the RAE was more significant for athletes selected for the National Junior Tennis Team, which had an OR of 2.19, and for which the percentage of players with birth dates in the first half of the year was 61.2%.

Overall, this study revealed differences in the effects of date of birth on the athletic performance of Chinese junior female tennis players. Athletes born closer to the beginning of the year were more likely to achieve excellent athletic performance in the lower age categories, whereas in these same categories, athletes born later in the year experienced greater difficulty achieving excellent results. However, this trend was found to decrease with age, and birth dates became largely irrelevant to athletic performance in the U16 category, with athletes born at the end of the year being just as likely to achieve excellence in athletic performance.

## Supporting information

S1 Data (XLSX)
